# A glimpse of the end-of-life of people with Parkinson's disease and atypical parkinsonism: A descriptive analysis of electronic health records

**DOI:** 10.1177/1877718X251372875

**Published:** 2025-09-02

**Authors:** Herma Lennaerts-Kats, Anke Elbers, Catharina Muente, Rebecca van Stigt, Bastiaan R Bloem, Kris CP Vissers, Marieke M Groot, Marjan J Meinders

**Affiliations:** 1 Department of Neurology, 6034 Radboud University Medical Center, Donders Institute for Brain, Cognition and Behaviour, Center of Expertise for Parkinson & Movement Disorders, Nijmegen, the Netherlands; 2 Attent zorg en behandeling, Arnhem, The Netherlands; 3 Department of Anesthesiology, Pain and Palliative Medicine, 6034 Radboud University Medical Center, Nijmegen, The Netherlands; 4 6985 Rotterdam University of Applied Sciences, Research Centre Innovations in Care, Rotterdam, The Netherlands

**Keywords:** Parkinson's disease, end-of-life, health services, hospice care, palliative care, euthanasia, palliative sedation

## Abstract

**Background:**

The needs of people with Parkinson's disease (PD) or atypical parkinsonism (AP) change significantly in the final weeks to days of life. A better understanding of this phase can help improve care.

**Objective:**

To examine healthcare use and end-of-life care in people with PD.

**Methods:**

We conducted a retrospective study (2022–2023) in three nursing homes, four hospitals, and eleven general practices in the Netherlands. Electronic health records of deceased individuals with PD or AP were reviewed for symptoms, healthcare use, and professional involvement.

**Results:**

We reviewed 189 records (70.4% PD; mean age 80.2; 68.1% male). In the last two weeks of life, patients had an average of 8.4 symptoms, with a higher burden in AP. Palliative sedation was used in 60.4%, most often in nursing homes (up to 78.3%) and among AP patients. Euthanasia occurred in 11 cases (6 PD, 5 AP), mainly in nursing homes and general practices. Antibiotics and pain medications were commonly used; fluid and oxygen therapy were more frequent in hospitals. Most patients were treated by a GP and 3–4 other healthcare professionals, but only 12.7% received support from a palliative care team.

**Conclusions:**

People with PD and AP face a high symptom burden at the end of life, yet palliative care involvement is limited. The frequent use of palliative sedation and cases of euthanasia reflect the complexity of this care phase. Better integration of palliative expertise and research into symptom management is urgently needed.

## Plain Language Summary

This study looked at how people with Parkinson's disease (PD) or atypical parkinsonism (AP) were cared for in the final phase of their lives in the Netherlands. This study involved a review of 189 electronic medical records from hospitals, nursing homes, and general practices. Most patients had PD and were, on average, 80 years old. In the last two weeks of life, they experienced an average of 8 different symptoms, such as fatigue, pain, difficulty swallowing, and reduced movement. People with AP tended to have more symptoms than those with PD. One major finding was the high use of palliative sedation—a treatment that reduces consciousness to relieve unbearable suffering. This was used in 60% of patients, especially in nursing homes and among people with AP. Euthanasia was performed in 11 cases. Other treatments included antibiotics and pain medication, which were frequently used. Hydration and oxygen therapy were more often given in hospitals. Most people received care from their general practitioner and 3 to 4 other healthcare professionals, with nurses involved in nearly all cases. However, only about 13% had support from a specialist palliative care team, which focuses on comfort and quality of life near the end of life. These results show that people with PD or AP often experience many difficult symptoms at the end-of-life, yet support from palliative care specialists is limited. The frequent use of palliative sedation suggests that more could be done to manage symptoms in other ways. More research and better coordination between PD specialists and palliative care teams are needed to ensure patients receive the best possible care in their final days.

## Introduction

Parkinson's disease (PD) is the most common form of parkinsonism, with a prevalence that is rising more rapidly than that of other neurodegenerative disorders.^1,2^ In addition to PD, the clinical syndrome of parkinsonism encompasses several atypical parkinsonian disorders, including multiple system atrophy (MSA), progressive supranuclear palsy (PSP), corticobasal degeneration (CBD), and dementia with Lewy bodies (DLB).^3^ Both PD and atypical parkinsonisms (AP) have a profound impact on patients, their families, caregivers, and society as a whole, leading to substantial physical, emotional, existential, financial, and social burdens.^4-6^

Currently, no curative treatment exists for PD or AP.^3,7^ In PD, disease progression often leads to complications such as aspiration pneumonia, falls, and urinary tract infections, which are common causes of death.^7^ Despite the availability of various symptomatic therapies, late-stage PD severely impairs emotional and social functioning as well as the ability to perform daily activities.^8-11^ AP typically progress more rapidly than PD and respond poorly to dopaminergic treatment.^3^ Individuals often present with symptoms such as frequent falls, autonomic failure, or cognitive impairment. These conditions are associated with a poorer prognosis, including shorter survival and more severe functional decline.^12^

A retrospective cross-sectional study of 1439 individuals in Canada (2011–2017), examined health care utilization in the last year of life among those with neurodegenerative movement disorders.^12^ Both PD and AP were associated with high acute care use, including frequent emergency department visits (>63% in PD; >70% in AP) and hospitalizations (>55% and >60%, respectively). ICU admissions were rare (<3%). Outpatient neurologist involvement was limited (36% in PD; up to 62% in AP), and palliative care use was low (7–17%). Most patients died in hospital or long-term care, with fewer than 10% dying at home. Early palliative care and extended home care were linked to a higher chance of dying at home.

Given the progressive nature of PD and AP, and the increasing complexity of care needs over time, timely access to palliative care is essential for patients and their families.^8,13^ As the disease advances, particularly in late-stage and end-of-life phases (defined as the last days, weeks, or at most a few months of life), a palliative approach becomes increasingly important.^12,14-16^ Special attention should be given for people with PD to oral dopaminergic medication, which is often discontinued due to swallowing difficulties or reduced consciousness. Non-oral alternatives, such as transdermal rotigotine and subcutaneous apomorphine, may help manage symptoms, although they carry risks such as confusion or nausea and are not always feasible.^17-21^ Decisions regarding continuation or tapering of medication should be guided by the patient's comfort, preferences, and the care setting. Furthermore, end-of-life-care requires an approach tailored to the individual receiving it, including taking special care to involve the patient's loved ones, managing complex and refractory symptoms, and paying attention to spiritual and existential needs. Evidence from other disease areas—such as dementia, cancer, and COPD—demonstrates that timely integration of palliative care can enhance patient autonomy, reduce non-beneficial interventions, and lower overall health care costs.^22-25^

Despite growing recognition of the need for palliative care, our understanding of the specific content and quality of end-of-life care in PD and AP remains limited. In particular, little is known about how care is actually delivered during the end-of-life, including the final days and weeks of life. This lack of insight hampers efforts to tailor care to the complex needs of this population. Therefore, the primary aim of this study was to map end-of-life care practices across different care settings for individuals with PD or AP, using retrospective electronic health record data.

## Methods

We conducted a retrospective study in nursing homes, hospitals, and primary care practices in the Netherlands, with the aim of capturing the diversity and context of care provided in each setting. Recognizing that differences in patient populations and documentation practices across these settings preclude direct comparison, our focus was to explore setting-specific patterns in end-of-life care for people with PD or AP. In total, we aimed to include 150 electronic health records, evenly distributed across the three care settings (approximately 50 per setting). The decision to review this number of records was based on the expert judgment and prior experience of the ParkinsonSupport project team in palliative care research.^8,26-35^ Although no formal power calculation was performed, this sample size was considered sufficient to identify meaningful patterns while remaining feasible for retrospective analysis. For additional context, Box 1 provides background information on the structure of the Dutch healthcare system, particularly regarding advance care planning and palliative sedation.

Box 1. Contextual overview of the Dutch healthcare system and end-of-life care practices.

The Dutch healthcare system is characterized by a coverage through mandatory health insurance, with an emphasis on primary care.^36^ General practitioners (GPs) serve as gatekeepers and are often the first point of contact, managing chronic illnesses and referring patients to hospital-based specialists when needed. Hospitals provide secondary and tertiary care, while long-term care is delivered through nursing homes and home care services under the Long-Term Care Act. Elderly care physicians play a central role in nursing homes.

Advance care planning (ACP) is an integral part of the system, particularly in the context of chronic and terminal illness.^37^ GPs and elderly care physicians frequently initiate conversations about future care preferences, including resuscitation, hospital admission, and end-of-life care. These discussions aim to align (medical) care with patients’ values and are documented in medical records.

Palliative sedation is defined as a treatment with sedative medications used to alleviate suffering from refractory symptoms of a patient with a terminal illness by reducing their consciousness. suffering.^38,39^ In the Netherlands, palliative sedation is governed by guidelines issued by the Royal Dutch Medical Association (KNMG).^40^ According to these guidelines, palliative sedation is permissible when a patient is experiencing unbearable suffering from refractory symptoms and has a life expectancy of two weeks or less. It is considered part of normal medical practice and does not require reporting to the authorities, unlike euthanasia, which is subject to strict legal procedures.

According to the Dutch guideline on palliative sedation (2022), midazolam is the first-line agent for all forms of palliative sedation in adults.^41^ Continuous sedation typically starts with a subcutaneous bolus, followed by a maintenance dose, adjusted as needed. If insufficient, levomepromazine can be added. In complex cases, escalation to propofol or phenobarbital may be considered in consultation with a palliative care team. In acute sedation, higher doses of midazolam are used, often combined with morphine for pain or dyspnea. Treatment is individualized to achieve proportional sedation and ensure patient comfort.

### Study setting and participants

We reviewed electronic health records of patients with a diagnosis of PD or AP who died within the past five years (October 2022 – November 2023). We contacted nursing homes and hospitals directly to request their participation, leveraging previous collaborations with neurologists, PD nurses, and elderly care physicians from an earlier ParkinsonSupport project.^32^ For the general practices, we used routinely collected data from 11 fully computerized general practices in the Radboudumc Family Medicine Network, covering over 66,000 patients in the eastern part of the Netherlands. In these participating practices, all medical ethical and privacy requirements have been met.^42^ In accordance with ethical and procedural guidelines, patients with a documented opt-out note in their medical record, indicating refusal to participate in research, were excluded from the study.

To systematically collect detailed quantitative and qualitative information about the last year of life, we developed a structured data extraction form. This form was informed by existing literature and data collection tools used in studies on end-of-life care in older patients and general practices.^43,44^ It was further refined through iterative development based on the first three reviewed records and pilot-tested independently by two researchers (AE and HL) using five patient files. Discrepancies were discussed and resolved to ensure consistency; no substantial interrater differences were found thereafter. Data were manually extracted from records of general practices, hospitals, and nursing homes, and entered into a unified database using a standardized format. The final data extraction form contained four different sections with the following elements:

1. Patient characteristics and general information: age at death, sex, comorbidities, cause and location of death, and survival in years from diagnosis;
2. Prevalence of symptoms in the last six weeks of life;
3. Medical consumption in the last six weeks of life: hospital admission, referral to hospice, medication use, other medical interventions (artificial feeding, fluid administrations, oxygen therapy) and end-of-life interventions (palliative sedation, euthanasia)
4. Health care use: number of contacts with specialists, allied health professionals, nurses, use of palliative care consultation team, identification of interventions.

Statistical analyses were performed with IBM SPSS Statistics 22. Descriptive statistics were used for all outcome measures. Qualitative responses to open-ended text boxes (interventions of healthcare professionals) were analyzed using inductive thematic analysis by two researchers (HL & AE). Verbatim information were first compiled in Atlas.ti 22.0. HL conducted the initial coding, focusing on responses to key question: which interventions were provided in the last six weeks before death by healthcare professionals? These responses were organized into preliminary thematic categories. Themes and codes were then discussed in meetings with AE, and any discrepancies were resolved through consensus. After this joint review, HL continued coding the remaining data, followed by a final consultation to refine and validate the themes. This iterative approach ensured consistency in interpretation and reliability of the findings for Table 4.

## Results

The selection procedures resulted in 189 medical records being reviewed, of which 58 were from five hospitals, 91 from three nursing homes and 40 from 11 primary care practices (Table 1). The mean age of the patients at the time of death was 80.2 years (SD 7.4) and 68.1% were men. The majority of patients (70.4%) were diagnosed with PD. The most common documented cause of death being infection (27.0%), followed by COVID (12.7%), frailty (12.7%) and cardiovascular disease (11.1%).

**Table 1.** Patient characteristics.

	Total (n = 189)	Hospital (n = 58)	Nursing home (n = 91)	General practice (n = 40)
Demographics				
Gender, n (%) male	130 (68.1)	45 (77.6)	58 (63.7)	27 (67.5)
Age at death in years, mean (SD)	80.2 (7.4)	78.3 (7.5)	79.9 (6.7)	83.4 (7.9)
Diagnosis, n (%)				
Parkinson's disease	133 (70.4)	40 (69.0)	68 (74.7)	25 (62.5)
Parkinsonism^a^	13 (6.9)	2 (3.4)	6 (6.6)	5 (12.5)
Progressive supranuclear palsy	10 (5.2)	2 (3.4)	5 (5.5)	3 (7.5)
Vascular parkinsonism	13 (6.9)	3 (5.2)	5 (5.5)	5 (12.5)
Multisystem atrophy	8 (4.2)	4 (6.9)	3 (3.3)	1 (2.5)
Lewy body dementia	4 (2.1)	2 (3.4)	2 (2.2)	0
Corticobasal degeneration	1 (0.5)	0	1 (1.1)	0
Other, multiple	7 (3.7)	5 (8.6)	1 (1.1)	1 (2.5)
Documented cause of death, n (%)				
Infection (including lung infection)	51 (27.0)	15 (25.9)	23 (25.3)	13 (32.5)
Frailty	24 (12.7)	6 (10.3)	13 (14.3)	5 (12.5)
Respiratory	9 (4.8)	4 (6.9)	3 (3.3)	2 (5.0)
Cancer	10 (5.3)	4 (6.9)	2 (2.2)	4 (10.0)
Cardiovascular	21 (11.1)	10 (17.2)	7 (7.7)	4 (10.0)
COVID	24 (12.7)	7 (12.1)	17 (18.7)	–
Fracture	8 (4.2)	5 (8.6)	3 (3.3)	–
Other^b^	26 (13.8)	7 (12.1)	13 (14.3)	6 (15.0)
Not applicable / unknown	16 (8.5)	–	10 (11.0)	6 (15.0)
Other clinical characteristics				
Disease duration in years, mean (SD)	6.4 (10.1)	6.3 (4.5)	6.9 (14.4)	5.8 (4.5)
Charlson comorbidity index, mean (SD)	4.9 (8.1)	4.5 (1.7)	4.4 (11.2)	6.8 (2.6)
Levodopa equivalent daily dose (LEDD) in mg/day, mean (SD)	707.3 (492.0)	702.4 (410.2)	735.5 (538.5)	NA
Number of medications, mean (SD)	10.9 (4.6)	9.5 (4.5)	11.9 (4.6)	10.3 (2.7)

NA: data not available.

^a^ Parkinsonism not otherwise specified/unclear.

^b^ Other: cirrhosis of the liver, dehydration, euthanasia, multi-organ failure, voluntary stopping eating and drinking, metabolic dysregulation.

Table 2 shows medical use in the six weeks before death. A minority of patients living in nursing homes (PD 5.9%, AP 13.0%) or receiving care through general practice (PD 44.0%; AP 26.7%) were hospitalized during the last six weeks. Antibiotics were frequently used, especially in hospitals (PD 67.5%; AP 72.2%), with lower usage in nursing homes (PD 45.6%; AP 43.5%) and general practice (PD 40.0%; AP 20.0%). Pain medication was administered to the majority of patients in hospitals (PD 82.5%; AP 94.4%) and nursing homes (PD 94.1%, AP 95.7%), while it was less frequently used in general practice (PD 52.0%, AP 46.7%).

**Table 2.** Medical consumption in the six weeks before death.

	Hospital		Nursing home		General practice	
	PD (n = 40)	AP (n = 18)	PD (n = 68)	AP (n = 23)	PD (n = 25)	AP (n = 15)
Healthcare use						
Hospital admission, n (%)	40 (100)	18 (100)	4 (5.9)	3 (13.0)	11 (44.0)	4 (26.7)
Referral to hospice, n (%)	–	–	5 (7.4)	–	1 (4.0)	2 (13.3)
Medication use, n (%)						
Antibiotics	27 (67.5)	13 (72.2)	31 (45.6)	10 (43.5)	10 (40.0)	3 (20.0)
Changes in PD drug treatment	16 (40.0)	6 (33.3)	22 (23.4)	4 (17.3)	7 (28.0)	2 (13.3)
Pain medication	33 (82.5)	17 (94.4)	64 (94.1)	22 (95.7)	13 (52.0)	7 (46.7)
Medication to suppress hallucination or delirium	17 (42.5)		31 (45.6)	7 (30.4)	5 (20.0)	3 (20.0)
Other medical interventions, n (%)						
Artificial feeding	6 (15.0)	2 (11.1)	2 (2.9)	2 (8.7)	1 (4.0)	1 (6.7)
Fluid administration	26 (65.0)	14 (77.8)	9 (13.2)	3 (13.0)	2 (8.0)	2 (13.3)
Oxygen therapy	23 (57.5)	12 (66.7)	23 (33.8)	5 (21.7)	3 (12.0)	–
End-of-life interventions, n (%)						
Palliative sedation	23 (57.5)	11 (61.1)	45 (66.2)	18 (78.3)	12 (48.0)	4 (26.7)
Euthanasia	–	–	6 (8.8)	–	3 (12.0)	2 (13.3)

Changes in PD-related drug treatment in the six weeks before death were noted in 16 (40.0%) of hospitalized PD patients, 22 (23.4%) of nursing home PD patients, and 7 (28.0%) of PD patients in general practice. These changes were often related to the presence of confusion, delirium, somnolence, agitation, difficulty swallowing, and increased rigidity. Adjustments included withdrawal, increase or decrease of antiparkinsonian medication and switching from oral to transdermal medication. A few cases reported the start of more invasive treatments such as apomorphine infusion or intrajejunal levodopa gel.

Fluid administration was a frequent medical intervention in hospitals (PD 65.0%, AP 77.8%), but was less common in nursing homes (PD 13.2%, AP 13.0%) and general practices (PD 8.0%, AP 13.3%). Artificial feeding was rarely used, with the highest occurrence in hospitals (up to 15%), and only incidental use in nursing homes and general practice settings (ranging from 2.9% to 8.7%). Oxygen therapy was rarely used in general practice (PD 12.0%, no use reported in AP), but more commonly administered in hospitals (PD 57.5%, AP 66.7%) and to a lesser extent in nursing homes (PD 33.8%, AP 21.7%).

Palliative sedation was frequently used across all care settings, with the highest rates observed in nursing homes (PD 66.2%; AP 78.3%). In hospitals, 57.5% of PD patients and 61.1% of AP patients received palliative sedation. Usage was lowest in general practice settings (PD 48.0%; AP 26.7%). Euthanasia was performed in 6 PD patients in nursing homes and in 5 patients (3 PD, 2 AP) in general practice, with no cases in hospitals. Diagnoses included PD, PSP, unspecified parkinsonism, and cancer.

Table 3 presents the prevalence of symptoms in the last two weeks of life among patients with PD and AP. On average, patients experienced 8.4 symptoms during this period. The most commonly reported symptoms across all settings were reduced physical activity (91.1%), fatigue (66.5%), pain (65.4%), and swallowing difficulties (63.9%).

**Table 3.** Symptoms in the last two weeks of life in people with PD or APD.

Symptom, n (%)	Hospital		Nursing home		General practice	
	PD (n = 40)	AP (n = 18)	PD (n = 68)	AP (n = 23)	PD (n = 25)	AP (n = 15)
Reduction in physical activity	39 (97.5)	18 (100)	68 (100)	22 (95.7)	16 (66.7)	11 (73.3)
Fatigue	35 (87.5)	15 (83.3)	51 (75.0)	15 (65.2)	6 (25.0)	10 (66.7)
Pain	24 (60.0)	14 (77.8)	54 (79.4)	19 (82.6)	9 (37.5)	6 (40.0)
Difficulty swallowing	30 (75.0)	13 (72.2)	47 (69.1)	16 (69.6)	7 (29.2)	9 (60.0)
Loss of appetite	25 (62.5)	10 (55.6)	54 (79.4)	3 (13.0)	7 (29.2)	4 (26.7)
Urinary incontinence	31 (77.5)	15 (83.3)	37 (54.4)	13 (56.5)	17 (70.8)	4 (26.7)
Stiffness	27 (67.5)	10 (55.6)	32 (47.1)	13 (56.5)	2 (8.3)	3 (20.0)
Breathlessness	21 (52.5)	7 (38.9)	35 (51.5)	8 (34.8)	9 (37.5)	2 (13.3)
Delirium	20 (50.0)	9 (50.0)	30 (44.1)	11 (47.8)	9 (37.5)	2 (13.3)
Daytime sleepiness	17 (43.6)	9 (50.0)	34 (50.0)	12 (52.2)	4 (16.7)	4 (26.7)
Anxiety	13 (32.5)	11 (61.1)	32 (47.1)	10 (43.5)	1 (4.2)	3 (20.0)
Constipation	14 (35.0)	6 (33.3)	28 (41.2)	12 (52.2)	6 (25.0)	3 (20.0)
Hallucinations	20 (50.0)	6 (33.3)	25 (36.8)	8 (34.8)	3 (12.5)	2 (13.3)
Sleep problems	11 (27.5)	4 (22.2)	20 (29.4)	10 (52.2)	4 (16.7)	–
Decubitus	4 (10.0)	3 (16.7)	25 (36.8)	12 (52.2)	–	–
Depression	6 (15.0)	4 (22.2)	20 (29.4)	3 (13.0)	6 (25.0)	1 (6.7)
Nausea	4 (10.0)	2 (11.1)	18 (26.5)	9 (39.1)	1 (4.2)	3 (20.0)
Faint	17 (42.5)	5 (27.8)	8 (11.8)	2 (8.7)	2 (8.3)	1 (6.7)
Diarrhea	6 (15.0)	–	13 (19.1)	7 (30.4)	2 (8.3)	2 (13.3)
Nycturia	3 (7.5)	3 (16.7)	16 (23.5)	6 (26.1)	1 (4.2)	–
Dizziness	8 (20.0)	3 (17.6)	10 (14.7)	1 (4.3)	1 (4.2)	–

In hospitals both PD and AP patients showed a high burden of symptoms. Reduced physical activity was reported in nearly all cases (PD 97.5%; AP 100%). Fatigue, pain, and swallowing difficulties were also common, with AP patients showing slightly higher prevalence of pain (77.8% vs. 60.0% in PD).

Symptom prevalence was also high in nursing homes. Pain was more frequently reported in AP patients (82.6%) than in those with PD (79.4%). A notable difference was seen in loss of appetite, reported in 79.4% of PD patients but only 13.0% of AP patients. Pressure ulcers (decubitus) were also more common in AP patients (52.2% vs. 36.8%).

Fewer symptoms were documented overall in general practices. Pain was reported in 37.5% of PD and 40.0% of AP patients, while pressure ulcers were not documented at all. Fatigue and swallowing difficulties were more commonly reported in AP patients (66.7% and 60.0%, respectively) than in PD patients (25.0% and 29.2%).

Table 4 shows the involvement of healthcare professionals in the last six weeks before death. A total of 30 different healthcare professionals were identified in the electronic health records. Each patient had contact with an average of 3 or 4 healthcare professionals at the end-of-life, in addition to their general practitioner. In almost all cases (94.2%), nurses were involved to provide a wide range of interventions. Physiotherapists, dieticians, speech and language therapists and PD nurses were the next most commonly involved. A minority of patients received care from spiritual caregivers (16.4%) or a palliative care team (12.7%).

**Table 4.** Involvement of healthcare professionals and their interventions during the last six weeks before death.

**Healthcare professionals**	**Total (n = 189)**	**Interventions**
**Primary care physician** **Elderly care physician** **Geriatrician** **General practitioner** **Internist** **Neurologist** **Other (see ‘Other healthcare professionals)**	93 (49.2) 26 (13.8) 40 (21.2) 8 (4.2) 4 (2.1) 18 (9.5)	- set and implement medical policy - seeking advice from other healthcare professionals - coordinating medical care - family discussions - guidance at the end-of-life - discussing a person's wish for euthanasia - carrying out euthanasia
**General nurses**	178 (94.2)	- assistance with ADLs (activities of daily living) - wound care - end-of-life care, comfort care - medical interventions, such as initiation of pump therapy/pump connection for palliative sedation - screening for COVID and urinary tract infections - managing behavioral changes such as anxiety and aggression - observing and monitoring the patient's health - supporting discussions with the patient and family - administer tube feedings
**Physiotherapist**	79 (41.8)	- clearing phlegm - mobilization (after surgery) - optimization of transfers - stimulation of physical activation levels. - (Improving) physical condition and trunk control - increase respiratory strength - breathing exercises - improving activities of daily living - providing a walking aid and instructions on how to use it
**Dietician**	64 (33.9)	- determine comfort diet - screening for (poor) nutritional status with MUST/SNAQ - increasing protein intake, supplementation - advice on weight loss - advice and assessment for tube feeding initiation - support with COVID related feeding problems - supportive nutritional care for pressure ulcers or energy loss
**Speech therapist**	59 (31.2)	- swallow observation - advice on fluid and medication intake - improving communication skills - advice to carers and health professionals involved in communication change - Pitch Lee Voice Treatment
**PD nurse specialist and nurse practitioner PD**	56 (29.6)	- evaluate and adjust antiparkinsonian medication. The most commonly reported was the initiation of transdermal routines for problems with swallowing oral medications. - providing guidance during the end-of-life - providing guidance to general nurses on difficult patient behavior - pressure ulcer care - monitoring PD symptoms - advice on advanced therapies such as apomorphine therapy, deep brain stimulation and intrajejunal levodopa gel infusion.
**Psychologist**	36 (19.0)	- help patients cope with: * psychotic symptoms; * depression and mood disorders; * anxiety and hallucinations. - assist general nurses in coping with: * challenging behavior; * refusing medication; * daily structure. - advising family carers on how to deal with the patient's behavior - talking about a person's wish for euthanasia - determining a person's capacity to make decisions - cognitive screening assessment - assist in determining daily structure
**Neurologist**	35 (18.5)	- determine the cause of functional decline - advice and adjustment of antiparkinsonian medication. The most commonly cited was the initiation of transdermal medication if there were problems swallowing oral medication. - medical policy in case of delirium - consultation if a patient was in another department in the hospital - treatment for muscle spasms - initiation of advanced treatment options such as pump therapy (Intrajejunal levodopa gel infusion) - primary care physician in case of a hospital admission at the department of neurology - providing information to GP in case of a person's wish for euthanasia
**Elderly care physician**	33 (17.5)	- advice and adjustment of antiparkinsonian medication. The most commonly cited service was initiation of transdermal medication for problems with swallowing oral medication. - providing bereavement support - providing advice when a patient is transferred to another ward in the nursing home/hospital - providing palliative and end-of-life care - carry out family counselling - discussing a person's wish for euthanasia - carry out euthanasia
**Other medical specialists**	31 (16.4)	- orthopedic surgeon -> Treatment of a hip fracture - psychiatrist -> accompanies patients struggling with suicidal tendencies, determines a person's capacity to make decisions - gastrointestinal hepatologist -> Follows patients starting tube feeding, swallowing problems - internist -> treatment of anemia - geriatrician -> advice and medical policy for patients with multiple problems, admission to geriatric unit, swallowing problems - cardiologist -> Treatment of patients with acute respiratory distress or heart disease anesthetist -> pain medication for a patient with a hip fracture dermatologist -> Treatment of pemphigoid trauma surgeon -> treatment of fractures after a fall, neck brace
**Spiritual caregiver**	31 (16.4)	- support in coping with end-of-life - accompanying a person's request for euthanasia - supportive conversations - praying, making or listening to music - offering the last sacraments or anointing of the sick - conversations on spiritual issues, distress and/or life questions
**Palliative team or consultant palliative care**	24 (12.7)	- advice on palliative sedation - helping and supporting the initiation of palliative sedation - supporting patients and carers during the end-of-life - treatment of dyspnea - advice on comfort care - assist with a person's request for euthanasia
**Pain team or pain consultant**	6 (3.2)	- guidance and support for starting pump therapy to reduce pain - celiac block
**Oncologist**	5 (2.6)	- care and treatment of patients with metastatic cancer - primary care physician in the event of admission to the oncology unit
**Case manager**	5 (2.6)	- care coordination
**Other healthcare professionals**	50 (26.5)	- specialized nurses, support in referral to other health care institution, wound care, decubitus care - occupational therapist, assistance with transfers, decubitus care, optimizing sitting position, assistance in organizing daily structure, assistance to general nurses in daily activities for a PD patient, providing audio books, setting up an ergonomic chair - social worker, family support, coping with depressive feelings - music and dance therapist, well-being support, listening to or making music, hydrotherapy - art therapist - activity supervisor - complementary health professional, massage

A qualitative analysis of the interventions offered to people with PD showed that some interventions overlapped with other healthcare professionals. For example, discussing a patient's wish for euthanasia could be taken up by the GP, but also by a psychologist, geriatrician, palliative care consultant or spiritual caregivers. Another common intervention was the adjustment of antiparkinsonian medication, especially when non-oral solutions to antiparkinsonian medication were needed. In these cases, neurologists, elderly care physician, geriatricians and PD nurses were often actively involved. The palliative care team was part of the team in cases where palliative sedation needed to be started.

## Discussion

Our findings show that medical interventions such as antibiotics, hydration, and oxygen therapy were more frequently administered in hospital settings, whereas their use was more limited in nursing homes and general practice. We hypothesize that this pattern reflects the prevailing treatment focus in hospitals, which are generally geared toward curative or life-prolonging care. Patients admitted to hospital often arrive with the intent or hope of stabilization or recovery, which may lower the threshold for initiating active interventions—even in the final phase of life. In contrast, care settings such as nursing homes or home-based care tend to emphasize comfort and symptom relief, particularly when death is imminent. This distinction likely shapes clinical decision-making and contributes to variation in end-of-life care practices.

Furthermore, hospitalizations were relatively rare in the last six weeks for patients living in nursing homes (PD 5.9%, AP 13.0%) or receiving care through general practice (PD 44.0%, AP 26.7%), suggesting that a majority remained in their primary care setting until death. Despite high symptom burden, only 12.7% of patients received specialist palliative care. Compared to McKenzie et al. (2022), who investigated health care utilization in the last year of life among people with neurodegenerative movement disorders in Canada, our study indicates lower acute care use in the final six weeks.^12^ In their study, over 60% of patients had at least one emergency department visit and more than half were hospitalized, with nearly 46% dying in hospital. By contrast, our findings show that the majority of patients in nursing homes and under general practice care were not hospitalized in the final weeks of life. This may reflect differences in health system organization, care culture, or access to palliative and long-term care resources.

Nevertheless, both studies highlight a consistent gap: the underutilization of specialist palliative care. Despite the high symptom burden observed in both cohorts, only 12.7% of our patients and 8.8% in the Canadian cohort received outpatient palliative care. These findings emphasize the need for better integration of palliative expertise into the care of individuals with PD and AP, particularly in the final phase of life.

Our study revealed that a significant number of patients with PD experienced adjustments or discontinuation of their antiparkinsonian medication in the end-of-life. In approximately 30% of cases, treatment was altered due to symptoms such as dysphagia, somnolence, confusion, and increased rigidity. These findings align with previous literature indicating that swallowing difficulties and decreased consciousness often necessitate the cessation of oral dopaminergic medication during the terminal phase.^17-21^

Emerging evidence suggests that non-oral dopaminergic treatments can be effective in maintaining patient comfort when oral intake is no longer feasible. Apomorphine, administered subcutaneously, has shown benefit in terminal care by alleviating motor symptoms and supporting patient comfort, even in advanced stages of PD.^17-19^ Similarly, rotigotine patches offer a practical alternative and have been used successfully at the end-of-life, though their efficacy may be limited in some cases and side effects such as confusion and hypotension must be carefully monitored.^20,21^ However, healthcare professionals reported struggling with the continuation of dopaminergic therapy when patients lose the ability to swallow.^29^ The successful implementation of these treatments requires specialist knowledge, typically found within neurology or specialized PD nursing. Often non-neurological professionals are unfamiliar with these therapies or feel uncomfortable initiating and monitoring them, which may lead to a default reliance on more familiar symptom management strategies, such as palliative sedation, and results in missed opportunities for optimal dopaminergic symptom control.

This may, at least partially, explain the strikingly high rate of palliative sedation observed in our study cohort—on average 60% of patients with PD or AP received palliative sedation, which is twice the national average in the Netherlands.^38-46^ While sedation is an appropriate and often necessary intervention in certain contexts, its frequent use in PD patients raises the question of whether it may sometimes be a substitute for specialized dopaminergic symptom management that is not initiated due to lack of expertise. Although our data do not establish a causal relationship, the limited use of non-oral dopaminergic therapies and the high prevalence of palliative sedation suggest a potential association that warrants further investigation. Future studies should explore whether timely involvement of PD specialists and structured guidelines on dopaminergic treatment at the end-of-life can reduce the need for palliative sedation and improve quality of life for patients with PD in their final days.

Many healthcare professionals – e.g., physiotherapists, nurses, occupational therapists – may be involved in the patient's care at the end-of-life. We found that, on average, patients had contact with 3 or 4 healthcare professionals at the end-of-life, in addition to their GP. A palliative care team was involved in the care of only 13% of patients, according to documentation in the electronic health records. Dutch guidelines state that general palliative care should be provided by all healthcare professionals,^47^ but previous studies have shown that many healthcare professionals do not feel equipped to provide palliative care to this specific population.^29^ Therefore, the integration of palliative care teams offers an opportunity to improve palliative care for people with PD. Studies of palliative care teams in cancer populations have shown numerous benefits, including improved quality of life, mood, survival, pain and anxiety.^48-50^ Palliative care also reduces costs, minimizes hospital admissions and improves satisfaction in a variety of populations.^49,51-54^ However, other models have been proposed to further improve the organization of palliative care in PD, including consultative palliative care teams, integrated palliative care programs, and complementary models such as primary palliative care, a mobile consultation team, an acute palliative care unit, and an outpatient supportive care clinic.^55,56^ In addition, telemedicine services and home visits have been shown to have the potential to increase access to palliative care services.^57,58^

Finally, this study highlights that euthanasia can be an option in the management of end-of-life care of PD and AP, a topic that has received limited attention in previous research. We identified 11 cases of euthanasia (six PD and five AP), with 10 cases citing the underlying neurodegenerative condition as the sole reason for the request. While discussions on euthanasia in PD and AP are relatively scarce internationally, emerging data from countries with legalized medical assistance in dying (MAiD) provide valuable insights. A retrospective analysis by Nübling et al. (2021) from a Swiss right-to-die organization identified 72 patients with parkinsonian disorders (PD = 34, PSP = 17, MSA = 17, CBS = 4), accounting for 7.2% of all assisted suicide cases.^59^ These patients originated mainly from Germany (41.7%), Great Britain (29.2%), and the US (8.3%). Predominant symptoms at the time of application included immobility, helplessness, pain, dysarthria, and dysphagia.

Additionally, a review of euthanasia and assisted suicide in neurological diseases indicated that neurodegenerative conditions, including PD, are reasons for such requests in countries like Belgium, the Netherlands, and Canada.^60,61^ These findings underscore the importance of understanding the complex medical and ethical considerations surrounding euthanasia in PD and AP. In our study, six different healthcare professionals were involved in the euthanasia process, reflecting the multidisciplinary nature of end-of-life decision-making. Given the limited data on attitudes toward euthanasia among individuals with PD or AP, further research is needed to explore this sensitive aspect of care.

### Limitations

The present study has some limitations that we acknowledge. First, this study is based on retrospective analyses. Therefore, we cannot interpret whether the observed associations between symptoms, their treatment and there causes of death reflect causality. As a result, we cannot draw conclusions about the most effective treatment for symptom relief. Second, the medical notes from all involved health professionals were embedded within an integrated electronic health record in hospitals and nursing homes, but this was not the case for general practices which only included notes from the GP. Overall, symptoms were mentioned less often in these latter records, probably because we did not have access to notes from the entire multidisciplinary team. A third limitation of this study is that we did not have access to death certificates, which makes the reported cause of death less reliable. In some cases, frailty was recorded as the cause of death because these patients experienced a cascade of complications without a single, clearly defined cause documented in the medical records. Lastly, the reported average of 8.4 symptoms per patient may underestimate the true symptom burden, particularly in individuals with advanced PD or AP. As this was a retrospective study, the analysis was restricted to symptoms documented by healthcare professionals during routine care. It is likely that not all symptoms were systematically assessed or recorded, and thus the actual burden may have been higher than reflected in the data.
